# Politics No. II

**Published:** 1885-01

**Authors:** J. B. Chandler


### POLITICS NO. II.

J. B CHANDLER.

In the midst of the presidential campaign we had a few words to write for the October number of the BISTOURY on the subject of Politics. The article was necessarily trammelled by the desire to avoid in a magazine like this anything having an appearance of party bias.

Our numerous readers comprehend voters whose affiliations or convictions lead them to advocate the claims of different candidates and different parties; hence the impropriety of assailing either side in a journal which aims at an independent treating of any subject under discussion.

Now, however, the battle is over and we have to point with pride to the quiet and peaceful acceptance of a verdict which nearly half of the citizens of this great country strove to reverse.

There are a few party organs which continue to mutter discontent and prognosticate evil because their party has lost its hold, and perhaps their efforts may serve to delay the country from a speedy realization of that prosperity which certainly awaits only a return of confidence.

This country is not going backward, its march is onward. A political cyclone may uproot one party and startle us who had thought its lease of power was perpetual, but when the storm has passed away, we shall find as heretofore that with a change of servants in the higher places, the people have gained, not lost.

The able and tried statesmen of all parties will yet remain "at the fore" to aid the new comers to do right or to prevent them from doing wrong. Why then keep trying to stir up strife or discontent? Why should those who fought for, or against, a Union, to which all now point with pride, strive to stir up sectional strife, North against South; East against West; Atlantic against Pacific? "Let us have peace."

Let us now in the lull of party strife, look about us as citizens of a great and glorious Republic, not as petty ward politicians; let us consider the lessons we have learned in the past two decades, and try what use we can make of them. There are many points of interest which are pressing themselves into notice.

The frequency of elections, their demoralizing effect upon the people and upon general business. This may be corrected by lengthening the term of office of not only the President but of most of the elective officers in the States. Such a course would probably lead to a more careful selection of candidates and a more earnest scrutiny, by the capable voter, into character and capabilities of those to whom he is to give his suffrage.

The present plan of electing a President and Vice President is not satisfactory, it does not always express the will of a majority of the people and it also is liable to jeopardize the election of a valuable candidate for President, because of the unpopularity of the nomination for Vice President. Recent events have taught us that the tenure of life of a President of the United States is not always governed by the wishes of the people.

With the change of party power comes up the fear of the rotation in office cry: "To the victor belong the spoils." Why have any spoils? We educate men for our army why not for civil service? When a man fitted to a post is found let him be retained during life or until promoted. If old age finds him at his post, pension him. If he betrays his trust, let the punishment be speedy and such as to be a warning to all. Vacancies of course will of necessity occur, and merit being equal, influence will probably be a factor in the appointment. But under such a state of affairs we should soon find that our elections for the higher offices would not be belittled by the struggles and schemes of place hunters.

Other questions of National policy with regard to elections are receiving the attention of thinking men.

Many, and not without reason, argue against the privilege granted to the ignorant and irresponsive and indeed the vicious and criminal (unconvicted) to vote and in most instances by their votes to counteract those of parties possessing a deep interest in the perpetuation of the institutions of the country and ability to understand and direct them.

I am not an advocate for female suffrage but unless there should be restriction put upon the elective franchise to correct the evil alluded to above, I do not see why a lady who pays taxes, and interests herself in the charitable institutions and works of her country should be excluded.

This morning I attended a sale of securities closing out estates &c., which is held weekly in Philadelphia and which draws together invariably a large number of capitalists; in the audience I noticed a number of ladies bidding upon bonds, making their own investments and conducting business on a large scale. How large a proportion of the voters at the late election could not do this. And if this is a mark of capability, why should women be excluded from the polls? I do not advocate woman suffrage because I see in it, what, to me, would take her from her sphere in life. But the time has arrived when some test should be enforced that would ensure the capability to understand the nature of a vote, and a direct interest in the perpetuation of the institutions of the country.

The last campaign was filled with lessons for even the professional politician. The people were early disgusted with the filthy personal attacks upon candidates. Crimination and recrimination. Again, the result did not seem to mark the approval of the people of a policy which brought the candidate for the high office of chief magistrate of the country into the arena in personal contact with the dirty work of an election campaign.

The last scenes in New York City in which all parties took part have unfortunately become history through the wide dissemination of the press. And many persons believe, not without some shadow of reason, that the great political contest for the election of President of the United States was decided by the effects of three alliterative words spoken by one whose vocation of life was not sacred from the invasion of political schemes.

In alluding to the errors of the campaign I have selected those which an unsuccessful result has proven futile; not from any desire to bring into prominence the faults of one party more than another but with the view of starting, if possible, the work of reformation in politics, or at least of arresting the downward progress and the unblushing and open use of means which are foreign to the true spirit of self-government and republican institutions.

I am aware that a very different view of causes and effects in considering the last election may be taken by able men of different political parties, the relative merits and popularity of the candidates, the advantages and disadvantages of a long or short career in statesmanship and other factors in argument, but the question is settled and there remain to the people the lessons of the past and the hopes of the future, it is but fair to allow the new administration the chance to do right, and to aid as far as possible in the restoration of confidence and unity of interests throughout the whole country.

See advertisement of the second edition of BODINES.

